# Building Bridges Between the Clinic and the Laboratory: A Meeting Review – Brain Malformations: A Roadmap for Future Research

**DOI:** 10.3389/fncel.2019.00434

**Published:** 2019-09-27

**Authors:** Tamar Sapir, Tahsin Stefan Barakat, Mercedes F. Paredes, Tally Lerman-Sagie, Eleonora Aronica, Wlodzimierz Klonowski, Laurent Nguyen, Bruria Ben Zeev, Nadia Bahi-Buisson, Richard Leventer, Noa Rachmian, Orly Reiner

**Affiliations:** ^1^Department of Molecular Genetics, Weizmann Institute of Science, Rehovot, Israel; ^2^Department of Clinical Genetics, Erasmus University Medical Center, Rotterdam, Netherlands; ^3^Department of Neurology and Neuroscience Graduate Division, University of California, San Francisco, San Francisco, CA, United States; ^4^Pediatric Neurology Unit, Fetal Neurology Clinic, Wolfson Medical Center, Holon and Sackler School of Medicine, Tel Aviv University, Tel Aviv, Israel; ^5^Department of (Neuro-)Pathology, Amsterdam Neuroscience, Amsterdam UMC, University of Amsterdam, Amsterdam, Netherlands; ^6^Stichting Epilepsie Instellingen Nederland (SEIN), Zwolle, Netherlands; ^7^Nalecz Institute of Biocybernetics and Biomedical Engineering, Polish Academy of Sciences, Warsaw, Poland; ^8^GIGA-Stem Cells, Interdisciplinary Cluster for Applied Genoproteomics (GIGA-R), C.H.U. Sart Tilman, University of Liège, Liège, Belgium; ^9^Sackler School of Medicine and Pediatric Neurology Unit, Edmond and Lilly Safra Pediatric Hospital, Tel Aviv University, Tel Aviv, Israel; ^10^INSERM UMR 1163, Imagine Institute, Paris Descartes University, Paris, France; ^11^Necker Enfants Malades Hospital, Pediatrric Neurology APHP, Paris, France; ^12^Department of Neurology, Royal Children’s Hospital, Murdoch Children’s Research Institute, University of Melbourne, Parkville, VIC, Australia; ^13^Department of Pediatrics, University of Melbourne, Parkville, VIC, Australia

**Keywords:** brain malformations, brain development, neuronal migration, neuronal progenitors, brain organoids, mouse models, mTOR, brain plasticity

## Abstract

In the middle of March 2019, a group of scientists and clinicians (as well as those who wear both hats) gathered in the green campus of the Weizmann Institute of Science to share recent scientific findings, to establish collaborations, and to discuss future directions for better diagnosis, etiology modeling and treatment of brain malformations. One hundred fifty scientists from twenty-two countries took part in this meeting. Thirty-eight talks were presented and as many as twenty-five posters were displayed. This review is aimed at presenting some of the highlights that the audience was exposed to during the three-day meeting.

## Evolution and Development – Spotlight on Progenitors

**FIGURE 1:**
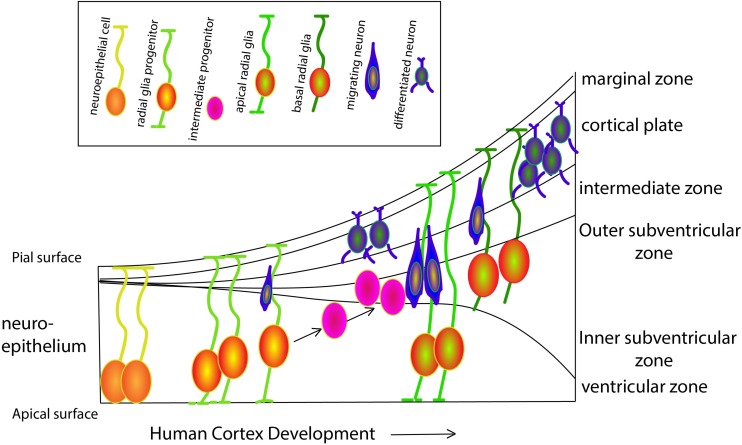
Schematic overview of progenitor populations during human cortex development. At gestation week three the neural tube appears. Neuroepithelial cells (NECs) that contact both the apical and pial surface divide symmetrically to exponentially amplify their number. While proceeding through the cell cycle, the nucleus moves toward the pial surface during G1-phase, undergoes S-phase close to the pial surface and then moves back to the ventricular surface during the G2-phase, where it will complete mitosis (M-phase) in a process known as interkinetic nuclear movements. Prior to neurogenesis, NECs lose the epithelial characteristics and differentiate to radial glial cells (RGCs) progenitors, that maintain connected with both the apical and pial surfaces. Until week six, RGCs self-renew by symmetric cell division, followed by asymmetric divisions generating one RGC and either a post-mitotic neuron, an intermediate progenitor (IP), or a basal RGC (bRGCs). bRGCs are located in the outer subventricular zone and are in contact only with the pial surface. These cells can divide asymmetrically to generate either neurons or IPs, playing a major role in human cortical surface expansion and folding (Sun and Hevner, 2014). All neurons generated by asymmetric division of RGCs, IPs and bRGCs migrate radially along the basal process of RGCs toward the pial surface, to form a transient structure called the preplate. The preplate includes the reelin-secreting Cajal-Retzius cells (later becoming cortical layer I) that settle right below the pial surface and are essential for the termination of cortical neuron migration. Later, neurons migrate toward Cajal-Retzius cells and settle in the cortical plate in an inside-out fashion to form the other six layers of the cortex.

Our cognitive capacity is housed in the complex and well-organized cerebral cortex. Although the basic principles, cellular mechanisms and a large proportion of the molecules that underlie cortical development are shared by mammalian species, the human brain has exceptional features that have been better understood in recent years, thanks to the ground-breaking work of several distinguished labs. Prof. Arnold Kriegstein and Prof. Wieland Huttner and colleagues have been among the pioneers of a trend of shifting from the classical rodent model systems and focusing on human cortical development. Kriegstein and his colleagues, study the progenitor cell population that shapes the developing human brain, in particular the population of progenitors that is responsible for neurogenesis of the upper layers in the human brain and resides in the outer subventricular zone (OSVZ) (see Figure 1 for an overview of the various cell types during human cortex development). This area was first described in the primate cortex (Smart et al., 2002), and was later shown to generate upper layer neurons (Lukaszewicz et al., 2005). OSVZ progenitors include transit amplifying intermediate progenitor (IP) cells and outer or basal radial glia (oRG/bRG) while ventricular or apical radial glia (vRG/aRG) reside in the ventricular zone. Delamination of vRG cells precedes transformation of vRG cells to oRGs. vRGs and oRGs form a discontinuous radial scaffold that spans the width of the developing cerebral cortex. This unique organization appears during mid-gestation approximately at the onset of neurogenesis of the excitatory neurons of layer 3 and 4. The new scaffold replaces the continuous vRG scaffold that is similar to the RG fibers network in the mouse and is present in humans at earlier stages during the birth of the excitatory neurons of deep layers 5 and 6. This discontinuous scaffold will guide the progeny of the transit amplifying progenitors that migrate to the superficial cortical layers. A third population, the truncated RG, makes contacts with blood vessels, and constitutes an additional class of human progenitors. Characterization of the molecular signatures and the morphotypic diversity of these populations has progressed rapidly due to advances in single cell RNA sequencing technologies. Such an analysis led to a better understanding of the cellular events that allowed the evolutionary expansion of the human cortex as well as revealed new facets of brain malformations such as lissencephaly and microcephaly whose study was mostly limited to rodent models. A dynamic cellular motility that occurs during mitosis has been described as typical to most oRG cells (Hansen et al., 2010; review Ostrem et al., 2017). Mitotic somal translocation (MST), is a rapid, basal-directed “jump” of the oRG soma prior to cytokinesis. Besides this unusual migratory behavior, oRGs display a unique transcriptional signature that consists of members of several classes including a group of extracellular (ECM) production genes. Changes in ECM production may reflect a potential to influence the proliferative niche (Fietz et al., 2012) and the physical properties of cellular environment permissive to the formation of folds, shown in work from Prof. Huttner’s lab (Long et al., 2018).

Work from the lab of Dr. Silvia Cappello sheds lights on the molecular link between ECM, oRG and cortical folding. Dr. Cappello and her team identified a secreted molecule that binds cell membrane bound receptors and is essential for the delamination of oRGs in human derived organoids. Similarly, its expression in the mouse brain shifts the progenitors to a more basal location and induces ectopic folds by remodeling of cytoskeletal elements like F-Actin and β-catenin. The team demonstrated that mutations in this gene are associated with congenital malformations and periventricular heterotopia (PH). The transition between basic science and the clinic is not always a straight forward one, but nonetheless fascinating. Transcriptome profiling of oRGs led to a better understanding of the origin of Glioblastoma multiforme, a primary cancer of the brain and one of the most lethal ones. oRG transcriptional signatures highly correlate with one of the cellular population identified within the tumor and indeed, cells that display MST migratory dynamics were identified in tumors. Moreover, a direct correlation between MST, the stem cell-like properties and tumorigenicity of the tumor cells was demonstrated.

The expansion of the neocortex had been attributed to the increased proliferative behavior of the oRG/bRG and this population is therefore an important research target. Analysis of the transcriptional data collected from over four thousand single cells derived from different developmental stages allowed for the identification of genes module and signaling pathways that are typical for oRGs (Nowakowski et al., 2017). Tracing the key molecular players in the evolutionary neocortical expansion has been done in parallel directions. These include the search for new modes of gene regulation, novel gene function, and the identification of human-specific or signaling modules that are enriched in oRGs (Heide et al., 2017). An example of a signaling pathway that is enriched in oRGs is, LIFR/STAT3 that was found to promote the self-renewal of oRG. This finding has been translated to an improved *in vitro* modeling system. The activation of the Hippo-Yap signaling pathway was also correlated with the proliferative capacity of oRGs in human and in the ferret (Kostic et al., 2019). The expansion of the mammalian cortex was also a result of attenuation of signaling modules. Reduced Robo1 and Robo2 signaling, and increased expression of Dll1 in radial glia cells shifted the balance from direct neurogenesis to the production of intermediate progenitors (Cardenas et al., 2018). Despite the contribution of oRG to the evolutionary increased proliferative capacity of the human cerebral cortex, apical progenitors (vRG) are likely to have an impact as well. Meticulous study of the cell cycle dynamics of human and chimpanzees in 3D organoids, revealed a cell specific lengthening of prometaphase-metaphase duration in humans vRGs, while other mitotic phases remain unchanged (Mora-Bermudez et al., 2016).

The human bRG population, like that of the macaque (Betizeau et al., 2013), displays a remarkable morphological heterogeneity that was recently described (Kalebic et al., 2019). The complex morphology was linked to the activity of a morpho-regulatory splice variant of PALMDELPHIN resulting in an increase in the number of cell processes emerging from basal radial glial cells. A link between bRG proliferation and morphology appears to be common to both human and non-human primates (Dehay et al., 2015; Kalebic et al., 2019). ARHGAP11B (11B) is a human-specific gene that was shown to enhance oRG generation and proliferation and to induce ectopic convolutions in the otherwise smooth mouse cortical surface (Florio et al., 2015). ARHGAP11B emerged in evolution after a partial duplication of the ancestral ARHGAP11A, a Rho guanosine triphosphate activating protein (RhoGAP). The duplicated gene underwent a substitution mutation that created a new splice variant which had lost 55 nucleotides, and gained a human-specific C-terminal domain (Florio et al., 2016). The novel protein product no longer possesses RhoGAP activity. A new study from Prof. Wieland Huttner’s lab, presented by Dr. Takashi Namba highlighted a continuous effort to understand ARHGAP11B contribution to human cortical expansion. Unlike the ancestral ARHGAP11A, ARHGAP11B shows a remarkable colocalizations with the cell mitochondria, a domain-specific effect on mitochondria metabolism and an overall augmentation of bRG proliferation. This study adds to the accumulating data that highlights mitochondria not merely as a supplier of energy but rather as a regulatory center of stem cell proliferation and differentiation (review Khacho and Slack, 2017).

Prof. Fiona Francis presented a study that focuses on the contribution of progenitors to brain malformations. Prof. Francis described a surprising involvement of a postsynaptic density protein, in subcortical heterotopia, polymicrogyria (PMG), pachygyria and global developmental delay. Prof. Francis found that the protein is expressed during mouse cortical development in multiple regions including the proliferative zones, the VZ and the SVZ. Its knockdown leads to changes in the spindle orientation of the progenitors, causing premature delamination of the RG and thus to increased abundance of intermediate progenitors. These changes were linked to downregulation of F-Actin at the ventricular surface. Further elucidation of the phenotype came from the identity of scaffolding protein partners that are present at the postsynaptic density as well as in the dividing progenitor.

## Migrating Cells – Not the End of the Road

Neuronal migration is a well described phenomenon that is essentially the movement of neurons from their place of birth to their final location in the brain. Decades of study led to the understanding that complex migratory events take place during cortical development which persists into the early postnatal ages not only in rodent but also in human (Sanai et al., 2011). In the mouse brain, excitatory neurons born close to the ventricular wall migrate in several modes. Early on, the neurons that will be destined to occupy the deep layers, will extend their leading edge to the outer most marginal zone and will pull themselves up, a behavior known as somal translocation (Miyata et al., 2001). Later, neurons that will delaminate from the ventricular zone will extend and retract processes regardless of their migratory trajectory in a mode of migration known as “multipolar migration” (Tabata and Nakajima, 2003). Multipolar migrating cells form transient glutamatergic synaptic contacts with the neurons that reside in subplate and these regulate the transition from a multi- to a bipolar morphology (Ohtaka-Maruyama et al., 2018). Following a transition to a bipolar morphology the cells will migrate in RG-dependent locomotion toward the pial surface. Just beneath the outermost cell-dense region of the CP, the primitive cortical zone (PCZ), cells will extend and retract their leading edge, a mode of migration that was thus named “terminal translocation”(Sekine et al., 2011). Prof. Kazunori Nakajima, a leader of a prominent research team, described a new type of migration which he rightfully named “erratic migration.” Cell that are “erratic” will not comply to the radial trajectories seen in most radially migrating neurons, as they will follow irregular paths and will change directions frequently. Lineage tracing based on photoconversion of KikGR was done in organotypic slice cultures with combination of *in vivo* genetically based tracing and revealed that these rogue cells are in fact glial progenitors that are born during prenatal stages. These observations led to the discovery of yet another migration mode; blood vessel guided migration. Prof. Nakajima and his team found that blood vessel guided erratic migration promoted angiogenesis and vessels branching. They were able to identify the molecules that mediate the attraction of the migrating progenitors to the endothelial cell wall, and validated their findings by loss of function studies using gene editing in both *in vivo* and *in vitro* systems. These finding may prove to be of an immense importance since glioma cells that are guided to blood vessels are likely to use an overlapping molecular mechanism.

The evolutionary expansion of the human brain was accompanied by acquisition of novel migration dynamics. Using Macaque as a non-human primate model the lab of Prof. Colette Dehay is studying the dynamic diversity of the OSVZ progenitors that produce the super granular layer compartment (SG). Prof Dehay found that the diversity of the molecular profiles of oRGs/bRGs is mirrored by the existence of cells with distinct morphologies (bipolar cells, cells with an apical process, as well as cells that have a transient apical or basal process). The bRG morphotypes differ in their neurogenic capacity, their progeny size and the directionally of the MST that can be either basally or apically directed. New levels of complexity of radial migration mode can be uncovered when lamina and area specific migration is carefully studied. Comparing SG neurons to deeper Infragranular layer (IG) migratory neurons shows higher radial migratory speed of the SG neurons prior to the appearance of the cortical convolutions. Areal difference in migration modes in area 17 and area 18 in the Macaque visual cortex were detected only for SG and not IG neurons, with a more directed radial movement in area 17 and more spread trajectories in area 18. These behaviors were attributed to cell autonomous changes in the leading-edge protrusions, and vesicular flow directionality that are controlled by differential levels of expression of molecular motor modulators in the different brain regions.

Neuronal migration follows either a radial or a tangential migratory pathway, depending on the area of the developing nervous system in which the neurons originate. Inhibitory interneurons follow a tangential migratory route, parallel to the surface of the ventricle. In the rodent brain, a saltatory type of tangentially migrating interneurons was described in which the leading edge bifurcates and the nucleus follows one of the branches while the other retracts. The molecular basis of the saltatory migratory pattern and its contribution to the timed invasion of the IN to the forming cortex, was until recently unknown. A recent manuscript from the lab of Prof. Laurent Nguyen (Silva et al., 2018) identified the molecular events and the importance of the non-continuous movement of interneurons. Carboxypeptidase 1 (Ccp1), the main glutamate chain deglutamylase, is found in the growth cone and around the nucleus of migrating interneurons. Its conditional removal from interneurons leads to protein hyperglutamylation and to a steadier, less saltatory migration. Ccp1 regulates actomyosin contractility by a novel enzymatic mode, acting on myosin light chain (MLC) kinase, an activity that is important to correctly time the interneurons invasion into the cortex. The untimely invasion of the interneurons to the cortex may result in an excitation/inhibition (E/I) imbalance in the cortical circuits which was suggested to be the undelaying mechanism in some neurodevelopmental brain disorders. The next challenge is therefore to assess the relevance of these finding in human conditions by harnessing novel modeling technologies such as brain organoids (see next section).

The lab of Prof. Mercedes Paredes is studying the developmental basis of brain malformation and operates under the working hypothesis that during the prolonged period of human brain development, cellular abnormal maturation may occur that will lead to aberrant connectivity and cortical dysplasia. Interneurons have an important function as coordinators of cortical activity, and as such are of great interest in studying the etiology of a large number of neuropathologies. Prof. Paredes, described a unique architectural organization of the proliferative niche of human interneurons, the hMGE that can re-form after xenografting. She suggests that one of the mechanisms that contributes to the rapid developing brain of the young infant is a collection of late arriving IN that migrate close the lateral ventricle and invade the frontal lobe (Paredes et al., 2016). This structure was named “Arc” and is in fact a transient medial migratory stream of late arriving interneurons that disappears after the first year of life. These findings change our view of the infant brain as being more dynamic than previously appreciated having active migratory streams that are targeting cortical regions that expanded in human. The postnatal developmental processes of the human brain may increase its vulnerability to insults from the environment.

Excitatory and inhibitory neurons are not the only cell type that exhibit impressive migratory ability. Cajal-Retzius cells (CRs), the first-born neurons in the developing cerebral cortex exhibit such activities. CRs are a transient population that consists of a collection of three linages that are born in distinct locations in the embryonic brain, all of which produce Reelin, an instructive ECM molecule whose function is critical for cortical lamination, and dendritogenesis (Ishii et al., 2016). Dr. Alessandra Pierani and her colleagues reported that the three CR linages display not only a unique birth and dispersion routes, but also disappear in a non-synchronous matter in a mechanism that is either bax-dependent or bax-independent (Ledonne et al., 2016). Dr. Pierani and her group, identified immature electrophysiological properties of the surviving CR cells and hypothesized that their persistence in the adult brain is connected to cortical dysfunction. To better understand Reelin contribution to cortical malformations, the effect of ectopic expression of several pathological Reelin variants was assessed. This system yielded region specific activities of the pathological variant that was consistent with the location and severity of malformations detected in patients.

## Brains in a Dish- Pushing the Limits

The introduction of human derived cerebral organoids jump-started our ability to mimic human specific cellular and molecular features of the developing brain that were until then largely inaccessible. Prof. Juergen Knoblich, and the group led by the late Prof. Yoshiki Sasai, established the first 3D brain organoids system and led the way to the widespread use of this model (Eiraku et al., 2008; Lancaster et al., 2013) (for reviews see (Di Lullo and Kriegstein, 2017; Arlotta and Pasca, 2019; Karzbrun and Reiner, 2019; Kyrousi and Cappello, 2019). This platform, is used by an increasing number of labs and received a considerable exposure in the current meeting. 3D organoids system has proven beneficial in studies on primate evolution, as well as studies of the cellular and molecular aspects of development and disease etiologies (review Kyrousi and Cappello, 2019). Dr. Silvia Cappello and her team were able to model Van Maldergem syndrome, a neuronal migration disorder caused by mutations in the receptor–ligand pair DCHS1 and FAT4 (Klaus et al., 2019). The cerebral heterotopias could be recapitulated *in vitro*, as evident by the presence of nodules of MAP2 + cells that were stacked close to the ventricles of the organoids. The migratory defect was in fact a sum of a dual contribution at the levels of the progenitors as well as that of the migrating neurons. Disrupted scaffold of the progenitors, was seen in the patient’s derived organoids as well as after acute, localized reduction of DCHS1 and FAT4 mRNA, due to destabilization of the cytoskeleton. This was accompanied by a shift in the transcriptome of vRG-like cells from patients-derived organoids to an oSVZ/iSVZ-like molecular signature. Mutated neurons exhibited cell autonomous changes in their migratory dynamics, with more resting time points and decreased velocity. This work not only dissected the molecular and cellular events that lead to the brain malformation and highlighted fundamental species-specific differences between mouse and human models. Knockdown of Dchs1 or Fat4 in the mouse led to a fundamentally different observation where within mouse embryonic neuroepithelium resulted in increased progenitor cell numbers and reduced neuronal differentiation (Cappello et al., 2013).

The strength of the organoid model in uncovering human specific cellular aspects of a migratory disorders is also evident in the work by Bershteyn et al. (2017). Reduced dosage of the *LIS1* gene product is the cause of isolated lissencephaly sequence (ILS) or the more severe Miller-Dieker lissencephaly Syndrome (Reiner et al., 1993). Organoids derived from patients’ cells, displayed an unusual MST phenotype revealing a human specific cellular feature in the disease mechanism. Lissencephaly oRGs translocated their soma to larger distances but either failed to divide or displayed a longer cell cycle. Actomyosin motors were found to be responsible for MST and their inhibition phenocopied the lissencephalic oRG behavior (Bershteyn et al., 2017).

Prof. Orly Reiner was able to model reduced gyrification, one of the hallmarks of lissencephaly. Prof. Reiner and her team were able to recapitulate the reduced folding formation in organoids derived from CRISPR-Cas9 edited hES lines with reduced levels of LIS1. An inherent change in the elasticity of the cells and the forces that drive folding, was detected along with transcriptional abnormalities that point to the extracellular matrix as an important contributor to the cellular environment that supports proper gyrification (Karzbrun et al., 2018).

Larger and more diverse intermediate progenitor populations correlates with a larger brain size. Interestingly genes that are thought to be involved in the evolutionary expansion of the human brain are often those whose loss of function leads to microcephaly or other brain malformation. Prof. Knoblich presented a pipeline for the genetic screening of microcephaly candidate genes, of which mouse modeling is often inefficient due to a subtle manifestation of the mutations.

Modeling Autism spectrum (ASD) disorder is a challenging task. ASD is a heterogeneous disease in multiple aspects; in its phenotypic manifestation and severity, in its genetic roots and in the complexity of contributing environmental factors. Dr. Gaia Novarino, undertook this challenge and is focusing on ASD caused by the loss of function of chromatin modulators. She is asking not only what is the etiology of ASD but also is there a particular developmental time window in which the gene function is critical and whether the deleterious effects may be reversible. Chromodomain helicase DNA binding protein 8 (*CHD8*) encodes a chromatin remodeling factor. Haploinsufficiency due to *de novo* mutations that span the entire CHD8 gene define a genetic subtype of ASD (Bernier et al., 2014). Prof. Novarino finds that the loss of function of CHD8 leads to unbalanced vRG divisions in the ventricular zone of mutated organoids or of mutated cells in chimeric organoids, leading to elongated and “thinner” ventricular zones and eventually larger organoids. Single cell RNA sequencing revealed hundreds of differentially expressed genes many of which are known to be linked to ASD. The transcriptional signature of the vRGs pointed at misregulation of cell cycle genes, an activation of a signaling pathway that potentially increases the surface area of the ventricular zone, but also suggested the involvement of the IN populations in the disease etiology.

Despite being extremely useful, 3D brain organoids possess inherent limitations. One obvious limitation is the inability to report for behavioral outcomes, which represent the clinical readout of most if not all brain disorders. In addition, while the human cortex contains hundreds of cell types with distinct transcriptional signatures, it appears that it may be challenging to represent all the cell types in organoid models. The spatio-temporal diversification and neuronal activity add layers of complexity to the fabric of cells that are building the human cortex (Mayer et al., 2019; Miller et al., 2019).

Cerebral organoids contain the main cell modalities that are found in the developing brain but their cell repertoire is simpler than that of a primary tissue of a comparable developmental stage (Pacitti et al., 2019). Organoids display an unpredicted anatomy and great heterogeneity partially due to lack of areal organization. Moreover, the large 3D organoids are hard to image in a non-disruptive manner and their size tends to limit nutrient delivery. Currently, each of these limitations is being approached experimentally or technologically. Approaches to increase the complexity of organoids include fusion of pre-specified organoids to a larger structure known as assembloid (review Arlotta and Pasca, 2019), and the engineering of an artificial cellular organizer that secrets instructive morphogens in a polarized manner (Cederquist et al., 2019). Nutrient penetration and visibility can be achieved by limiting the growth of the organoid in a confined semi-transparent space (Karzbrun et al., 2018), by constant stirring (Qian et al., 2018) or by growing organotypic slices on an air liquid interface (Giandomenico et al., 2019). Alternatively, the formation of vascularized organoids can be achieved by coating the organoid with iPSC derived endothelial cells (Pham et al., 2018) or by transplantation of human derived organoids into an immune deficient mouse brain (Mansour et al., 2018). Therefore, mouse models still remain extremely useful for studying a large number of brain malformations and are often used in conjunction with 3D organoid models.

## Malformations of Cortical Development (MCDs): Classification, Diagnosis and Treatment

With the accessibility of more advanced imaging, it has become apparent that MCDs are heterogeneous, with distinct characteristics, extent, and location.

In the past, malformation such as agyria/pachygyria could not be diagnosed before 22 weeks of gestation due to the smooth appearance of the cerebral hemispheres, which hampered early detection by imaging. Fetal brain malformations may remain undiagnosed genetically since they might not have a yet detectable genetic etiology, or they are the result of a somatic mutation that cannot be detected by standard prenatal genetic screens, or are not genetic. Usually these severe malformations remain undiagnosed since they might not be diagnosed by the time that US screening is performed even when using standard prenatal genetic diagnostic tests. Nevertheless, early diagnosis is obviously extremely important in order to enable parental counseling regarding the neurodevelopmental outcome. Prof. Gustavo Malinger, Dr. Karina Krajden Haratz, and Prof. Tally Lerman-Sagie demonstrated that in some cases and using high resolution transvaginal neurosonography techniques these conditions can be diagnosed at a very early gestational ages (13–20 weeks of pregnancy). The malformations that can be detected include periventricular nodular heterotopia, abnormal cortical layering, abnormal sulcation associated with polymicrogyria, corpus callosum agenesis, and microcephaly. Prof. Malinger and Prof. Lerman-Sagie added to the standard ultrasound examination the use of orthogonal planes and preformed meticulous search for detrimental findings. Significant signs that can be detected early include delayed cortical development, premature abnormal sulcation, hemispheric asymmetry, irregular shape or asymmetry of the lateral ventricles, thick or non-continuous cortex, irregular ventricular borders, corpus callosum abnormalities and abnormal interhemispheric fissure. Prof. Tally Lerman-Sagie also described the pathology features of dysgyria in a fetus with a tubulinopathy.

Cortical malformations classification schemes have been proposed to define the histological features of MCD. However, the complexity of the malformations is often more diverse than initially estimated using standard *in utero* imaging (Malinger et al., 2007).

Prof. William Dobyns presented a plethora of examples in which the current classification is partial or insufficient as it fails to express the complex pathologies seen in a single patient. He used polymicrogyria as an example of how the interpretation changes according to advanced insights. A more comprehensive classification scheme based on pathologic features as well as developmental and genetic etiologies is becoming useful as the new causative genes and deleterious mutations are being identified as the underlying causes for MCD (Barkovich et al., 2012). Dr. Grazia Mancini, described an effective evaluation process to identify the etiology of a large cohort of MCD patients. The evaluation process include accurate classification based on neuroimaging analysis, detailed family history and a search for gestational insults as possible non-genetic causes of the malformation. Dr. Mancini and her team were able to link monogenetic mutations, chromosomal anomalies and copy number variations to patients presenting various MCDs including microcephaly, lissencephaly, pachygyria, subcortical band heterotopia, cobblestone-type lissencephalies, polymicrogyria (PMG) and schizencephaly (de Wit et al., 2008; Meuwissen et al., 2016; Oegema et al., 2017; Dobyns et al., 2018; Vandervore et al., 2019). In this process, the availability of next generation sequencing tools, open access databases allowing exchange of variants and the multidisciplinary interaction at local and international level have proved great effectiveness.

Prof. Ute Hehr, from the University of Regensburg, leads an “open exomes” initiative for the identification of causative genes in a large cohort (approximately 350) of brain malformation patients. About third of the sequence variants that Prof. Hehr and her team identified across different brain abnormalities can be classified as pathogenic variants yet a similar proportion are of unknown significance. Prof. Hehr described two pathological synonymous variants in F-actin-binding cytoplasmic cross-linking phosphoprotein Filamin A (*FLNA*) in patients with Periventricular Nodular Heterotopia (PNH) in which the amino acids sequence of the predicted protein is not expected to change and speculates that the mutation may lead to mis-splicing of FLNA.

Prof. Nataliya DiDonato presented the concept of “Reverse Phenotyping” which often assists her in pointing at the correct genetic cause of particular MCD. Reverse phenotyping takes into consideration the refinement of phenotypes on genetic data (Schulze and McMahon, 2004). She describes the identification of phenotypes that can be recognized as a hallmark of a particular causative gene. Often such a phenotypic display is very informative when a complex genetic finding is presented and thus can assist with the diagnosis, the prognosis of a particular disease as well as in providing a useful genetic counseling.

The search for the genetic basis of brain malformations is not limited to protein coding gene. Dr. Stefan Barakat and his colleagues developed a genome wide unbiased and quantitative approach to identify functional enhancers, which it is currently being directed toward brain malformation, using 3D organoid disease models. Barakat’s protocol named “ChIP-STARR-seq,” is based on immunoprecipitation of chromatin using antibodies against transcription factors or histone modifications. This captures DNA sequences enriched for putative enhancers, that can be tested for enhancer activity in a massively parallel-reporter assay. To this end, DNA sequences are cloned *en masse* in the 3′ UTR of a reporter plasmid, that consists of a minimal promoter that drives eGFP expression. Upon cell transfection, plasmids that have integrated a functional enhancer will produce eGFP mRNAs, and since the DNA sequence was cloned upstream of the polyadenylation signal, the enhancer will also be transcribed. Therefore, RNA-seq of GFP positive cells and sequencing of plasmid DNA can be used to generate genome-wide enhancer activity maps that pinpoint to functional non-coding genome parts (Barakat et al., 2018). This approach enabled Barakat and his team to differentiate between a pancreas specific and a brain specific enhancer in a complex syndrome that involves both organs.

A relatively large variety of Long non-coding RNA (lncRNA) are expressed in the brain but their function in particular in the neurodevelopmental context is poorly understood. Dr. Igor Ulitsky and his team found specific lncRNAs that are required during neurogenesis. Additionally, they identified a conserved lncRNA that is located upstream to a chromatin modifier, *CHD2* (Chromodomain helicase DNA binding protein 2) a gene that is implicated in early-onset epileptic encephalopathy, developmental delay and autism. Complete deletion of the lncRNA promoter is embryonic lethal and a single allele removal increases the expression of *CHD2* due to a *cis* inhibitory effect of the lncRNA on CHD2 expression. This observation opens a possibility to augment the normal CHD2 allele expression levels in cases of *CHD2* haploinsufficiency (Rom et al., 2019).

MCD classification schemes are based on causative genes, include for example Tubulinopathies (caused by mutation of *TUBA1A*, *TUBB2A*, *TUBB2B*, *TUBB3*, *TUBB5*, or *TUBG1*) and Dyneinopathies (caused by mutation in the heavy chain 1 of cytoplasmic dynein, *DYNC1H1*). Prof. Nadia Bahi-Buission showed that despite being grouped together, each class actually contains a wide and often non-overlapping range of brain malformations. One of the challenges that basic research is facing, therefore, is not only discovering new causative genes but also understanding the often-complex genotype-phenotype correlation.

Connecting Actin genes (*ACTB, ACTG1*) variants to disease phenotypes, the best-known being Baraitser-Winter Cerebrofrontofacial (BWCFF) syndrome, was presented by Prof. Daniela Pilz. BWCFF is associated with cortical malformations, mainly pachygyria, in 60–70% of patients. However, mutations in *ACTG1* are also associated with isolated non-syndromic deafness and one has been reported in isolated coloboma. Variants in exons 5 and 6 of *ACTB* have recently reported with syndromic thrombocytopenia (ACTB-AST), and ACTB haploinsufficiency has been described with an ID syndrome associated with cardiac and renal malformations. A specific ACTB variant (c.547C > T; p.Arg183Trp) has been reported in Dystonia-Deafness syndrome (Cuvertino et al., 2017; Rainger et al., 2017; Yates et al., 2017; Latham et al., 2018; Skogseid et al., 2018).

Another suggested classification scheme, relies on a common denominator that is not necessarily the result of single causative gene but rather a common molecular or cellular event. Prof. Jim Barkovich described the use of MRI to study Dystroglycanopathies (Martin, 2005). This group of malformations are the result of discontinues glia limitans-basement membrane due to disturb attachment of oRGs to the pia may be caused by mutations in different genes; including *Laminin, GPR56, LARGE, COL4A1* and *Dystroglycans*.

Genetic classification may not apply to all cases of MCD. A reminder that brain malformation may not have a genetic etiology came from several speakers. Non-genetic causes include vascular abnormalities, described by Prof. Bruria Ben Zeev that can lead to hypoxia, or hypoperfusion as well as other common insults including infections of cytomegalovirus and Zika virus. Vascular abnormalities leading to focal brain malformations may also result from somatic and germ line mutations in genes such as GNAQ and ENG responsible for Sturge-Weber syndrome and hereditary hemorrhagic telangiectasia [HHT] syndromes, respectively.

The refinement and the reproducibility of the diagnostic criteria of brain malformations is one of the challenges which the clinicians are currently facing, requiring a multilayer comprehensive diagnostic approach that combines clinical, radiological, histological, and molecular genetics characteristics, according to Prof. Eleonora Aronica.

Novel digital pathology methods for computer-aided analysis of images were proposed by Prof. Wlodzimierz Klonowski. Applicability and usefulness of these methods have been demonstrated in analysis of histopathological images for cancer diagnosis (Klonowski et al., 2010, 2018). While traditional evaluation of tissue images by pathologists lead to interobserver differences and even intraobserver variations in the assessment, these computerized methods provide simple, fast and reproducible results. These methods can be adapted for quantitative MRI to improve MCD diagnosis.

While striving for an improved diagnosis using existing imaging technologies, a continuous attempt to develop new imaging technologies is underway. Prof. Igor Meglinski is developing a low cost, quantitative and non-invasive laser-based imaging platform. His team is trying to detect functional changes within the tissue, by following changes in the blood flow, aiming at the identification and classification of the malformation. Technological advancements are also harnessed for the development of effective treatment paradigms for children dealing with cognitive disabilities. Prof. Naomi Josman, from the University of Haifa shared the uses of Virtual Reality (VR) for the evaluation and treatment of the patients’ everyday function and quality of life.

## mTor Takes a (Second) Hit

The concept that deregulation of a signaling cascades is pathogenic and likely responsible for a wide spectrum of brain malformation echoed in the current meeting.

The mammalian target of rapamycin (mTOR) is a protein complex that is a member of phosphoinositide 3-kinase (PI3K)-related protein kinase family. The mTOR signaling pathway was highlighted in several talks as key to understanding both normal developmental sequences and pathological brain malformation. The mTOR pathway is an established role in multiple basic cellular events and its activation or inhibition is known to have harmful consequences (review Crino, 2015). Mounting evidence suggests that mTor is an important regulator of neuronal development and is involved in a number of malformations which are often referred to as “mTORopathies.” Mutations in mTOR regulatory genes (*TSC1, TSC2, AKT3, DEPDC5*) are associated with focal MCD, epilepsy and autistic traits in tuberous sclerosis complex (TSC) patients, hemimegalencephaly (HME), several megalencephaly (ME) subtypes, ganglioglioma (GG), polyhydramnios-megalencephaly-symptomatic-epilepsy (PMSE), familial focal epilepsy with variable foci (FFEVF) and cortical dysplasia) (Baybis et al., 2004; Miyata et al., 2004; Crino et al., 2006; Ljungberg et al., 2006; Dibbens et al., 2013). Prof. Eleonora Aronica presented an overview on the field as well challenges in identification and classification of the patients and described routes for treatments. A spatiotemporal analysis of gene expression patterns in Tuberous Sclerosis Complex (TSC) a prototypic monogenic disorder of mTOR pathway dysregulation which provides the rational mechanistic basis of a direct link between gene mutation and brain pathology (structural and functional abnormalities) associated with a complex clinical phenotype including epilepsy, autism and intellectual disability (Mills et al., 2017; Curatolo et al., 2018; Muhlebner et al., 2019). The TSC transcriptomic network is characterized by increased expression of genes associated with inflammatory, innate and adaptive immune responses and extracellular matrix organization (Mills et al., 2017). mTOR hyperactivation due to somatic mutations in affected regions contribute to the Focal cortical dysplasia type II (FCDII) and causes intractable epilepsy (Lim et al., 2015) (reviewed by Marsan and Baulac, 2018). Single-neuron genomics revealed high prevalence of somatic mutations in the brain with the estimated frequency of approximately 0.2 events per cell (Cai et al., 2015; Evrony et al., 2016). The contribution of local mTOR hyper activation due to mosaic *de novo* somatic mutations was suggested to be the underlying cause of HME (hemimegalencephly) lesions (Lee et al., 2012).

Prof. Stéphanie Baulac provided evidence of a somatic second-hit mutation in the mTORC1 repressor DEPDC5 pointing at a double-allelic hit: a brain somatic mutation identified in the postoperative specimen in addition to a germline mutation that sustain the focal nature of the cortical dysplasia (FCD) (Ribierre et al., 2018). An FCD-like phenotype could be recapitulated in a mouse model of focal and mosaic inactivation of Depdc5. Using a sequencing panel of mTOR genes, she was able to identify somatic mutations in mTORC1 pathway genes in >60% of FCD II cases (Baldassari et al., 2019). The hallmarks of FCD type II are dysmorphic neurons (type IIa), and large cells that are known as balloon cells (type IIb) and is a prevalent cause of refractory epilepsy (Blumcke et al., 2011). She showed these pathological cells carry the mutations (unpublished data). The second hit mechanism does not necessarily involve a germline mutation as seen in the work presented by Cristiana Pelorosso, form the lab of Renzo Guerrini. Pelorosso described an HME patient in which both causative mutations are somatic, and both appear in different genes of the mTOR pathway. She analyzed brain tissue from a patient that underwent surgery due to intractable epilepsy and found that the double mutant cells were not typical balloon cells, but that they are cytomegalic and display an aberrant electrophysiological activity. Pelorosso and her colleagues found that the combination of the mutant variants has a synergistic effect when expressed ectopically in embryonic rat brain, despite affecting different aspect in the developing cortex (NPCs proliferation and neuronal migration delay).

## MCD and Epilepsy-Open Questions

Epilepsy is prevalent in cases of brain lesions. It is common to heterogeneous disorders with a diverse and often unknown underlying genetic etiology. Prof. Renzo Guerrini presented the challenges in identifying phenotypic similarities using neurophysiological and imaging diagnostics (for reviews please see Guerrini, 2005, Guerrini, 2006; Guerrini and Noebels, 2014; Guerrini et al., 2014; Barkovich et al., 2015; Mei et al., 2017).

A/Prof. Richard Leventer, presented data that found that the dysmorphic neurons in the tubers of tuberous sclerosis appear to be denser in the tuber center compared to the tuber rim, which is consistent with recent work suggesting the seizures are generated from the tuber center. Additionally, A/Prof. Leventer, finds evidence for the second hit hypothesis. Deep sequencing of focal cortical dysplasia type IIA removed from a patient with a germline DEPDC5 pathogenic variant allowed the identification of a *de novo* somatic pathological variant of *DEPDC5*. The mutation gradient hypothesis, suggests that the epileptogenic centers display the highest density of somatic mutations, the higher the mutation load, the greater is the epieptogenicity (Mirzaa et al., 2016). Indeed, A/Prof. Leventer finds evidence that support this hypothesis. The allele frequency of second hit somatic mutations varies, with the highest mutation load correlating with the origin of the epileptic events and dysmorphic neuron cell density. A laser microdissection study, shows that the somatic second hit mutations are limited to the dysmorphic neurons in the lesion and are not present in normal appearing nearby neurons.

Studies that are aimed at understanding the causality between epilepsy and heterotopic lesions using animal models, were presented by Prof. Carlos Cardoso and Dr. Jean-Bernard Manent. FLNA is thought to influence neuronal migration and disruption of this process was suggested to be the main cause for periventricular nodular heterotopia (PNH) (review Guerrini and Carrozzo, 2001). Prof. Carlos Cardoso presented data showing that this may not be a sufficient mechanism to explain all aspects of the malformation. Prof. Cardoso and his team, find novel effects of FLNA during cortical development, not only on migrating neurons, but also on radial glia morphology and proliferation (Carabalona et al., 2012). Additionally, conditional knockdown of FLNA in mature neurons reveals mis-organization of the cortical network. Cardoso and his team were able to recapitulate epileptic seizures by locally knocking down FLNA in mouse models and described modification in the dendritic morphology and in the inhibitory input that the aberrant cells receive. By attributing the epileptic event to cortical neurons that are properly localized, Prof. Cardoso explains the relatively low correlation between the extent of Heterotopia in the patients and epilepsy severity.

Dr. Jean-Bernard Manent investigates the brain circuit defects which lead to epilepsy in animal models of subcortical band heterotopia. Manent and his group created a rat model of bilateral SBH by combining RNAi-mediated knockdown of DCX and tripolar *in utero* electroporation. These rats with bilateral SBH show spontaneous epilepsy that start at 2–3 months of age, and seizures become more frequent and last longer as epilepsy progresses (Sahu et al., 2019). This model allows Dr. Manent and his group to study how the size and position of band heterotopia differentially impact cortical circuit formation and organization in the overlying cortex, and predispose to epilepsy (Plantier et al., 2018).

## Morphology Function and the Plastic Brain

Finally, how well can prenatal diagnosis predict the pregnancy outcome? Prof. Daniela Prayer raised the possibility that separate developmental routes can lead to a common desirable outcome and thus she is challenging the notion that morphology predicts function. Prof. Prayer, points at mechanisms that are key to developmental plasticity namely, epigenetic modifications that can mediate between environmental factor and the levels of gene expression, and phenotypic plasticity, namely, the ability of a genome to produce different phenotypes.

Prof. Prayer and her team use both structural MRI and diffusion MRI to learn about the connectivity between different regions of the prenatal brain. In addition, they perform resting state fMRI and follow the function of different brain regions. Using these technologies, combined with a long term follow up of the diagnosed patients, Prof. Prayer presented case studies of fetal brain malformations that had variable and often surprising outcomes, ranging from normal development to severe developmental impairment.

Resting state functional MRI recapitulates known findings regarding the extent in which activity is preserved in lesion regions which can be remarkably high. Activation within morphologically abnormal regions is seen in majority of cases of polymicrogyria (PMG), Schizencephaly and FCD I and in a large proportion of FCD II and heterotopia cases (Janszky et al., 2003). Short range connectome studies reveal evidence not only for the preservation of the original activity in heterotopic, “double cortex” and unilateral PMG lesions but also the presence of ipsilateral connectivity and can predict preservation of motor functions (Foesleitner et al., 2018). Prof. Prayer names some of the landmarks that may assist the clinicians to improve their predictive capabilities in cases of Corpus Callosum Agenesis (CCA). The first is the extent of the malformation. An isolated CCA might predict a better developmental outcome. In cases of, isolated CCA remnant CC is most likely to be beneficial. Some cases of isolated CCA might present no connectivity across the hemispheres, however, their centrality may move to the temporal lobe. While, Colpocephaly (enlargement of the lateral ventricles) reduced hippocampal size (Knezovic et al., 2019) and abnormal lamination in other cortical regions may be indicative to a less desirable outcome.

## Future Directions

The last part of the meeting concerned an open discussion on major themes and points raised during the three days and focused on the issues: (1) understanding brain development in humans: what we know and what is still missing; (2) The issue of MCDs classification: what are the optimal future directions to integrate clinical, imaging, neuropathology and molecular data, and (3) the place of MCD animals models, i.e., to identify what is available, eventually through construction of a database and what is the place of alternative models (organoids, iPSCs, transdifferentiated neuronal cells).

One of the innovative aspects of this meeting was to gather clinicians and scientists in the field and try to get them to speak the same language and identify common research goals. The clinicians in the audience identify as possible threats to research development the lack of a common registry of MCD cases through European countries, the need to improve brain imaging in clinical setting, to define which types of follow up sequential imaging are needed and how to organize transition care and long term follow up studies. With advent of prenatal genome-wide analysis made available on large scale, areas which need to be further developed and integrated in the routine clinical practice are the prenatal with the postnatal brain MRI imaging or any other imaging technologies enabling accurate prediction of the phenotypes.

Basic scientists see as a threat to discovery of genes and mechanisms involved in MCD the fact that through NGS too many variants and too many genes are discovered, i.e., the tremendous heterogeneity of the molecular processes leading to brain malformations and the lack of a standardized way to interpret genomic variants. Hence, at the moment it does not seem possible to create a uniform pipeline for functional validation of variants, which requires specific approaches for each type of gene or pathway. Many are strongly in favor of linking basic science to clinic and apply discoveries achieved in animal models to human disorders in a more structured way. However, there is yet no unanimity about which model represents the best choice. The use of the ideal closest model to humans, the primate brain raises too many ethical issues to be considered as valid choice, while *in vitro* systems with human cells seem to progressively replace the role and overcome the limitations of mouse models. In this respect the creation of tissue banking and the way to connect these facilities would greatly facilitate research. Follow-ups on this meeting can be seen in the activities of COST Action CA16118; www.neuro-mig.org.

## Concluding Remarks

As summarized above, during this 3-day meeting a broad variety of talks related to basic and clinical research on MCD were presented. In an inspiring environment, fruitful discussions between clinicians, neuroscientists, geneticists, gynecologists, pathologists and other specialists took place that we feel will be the starting point for many collaborations and will enable further future advancements in the field that ultimately will lead to benefits for affected individuals and their families.

## Data Availability Statement

The datasets generated for this study will not be made publicly available. This is a meeting report and some of the speakers presented unpublished data. The text is confirmed by the speakers.

## Author Contributions

TS wrote the full outline of the review. TB edited and contributed the figure. MP, TL-S, EA, WK, LN, BZ, NB-B, RL, NR, and OR edited and formatted the manuscript.

## Conflict of Interest

The authors declare that the research was conducted in the absence of any commercial or financial relationships that could be construed as a potential conflict of interest.
